# Impact of Human Immunodeficiency Virus Drug Resistance Mutations Detected in Women Prior to Antiretroviral Therapy With Efavirenz + Tenofovir Disoproxil Fumarate + Lamivudine (or Emtricitabine)

**DOI:** 10.1093/ofid/ofae383

**Published:** 2024-07-15

**Authors:** Ceejay L Boyce, Tatiana Sils, Ross S Milne, Jackson J Wallner, Samantha R Hardy, Daisy Ko, Annie Wong-On-Wing, Malia Mackey, Nikki Higa, Ingrid A Beck, Sheila M Styrchak, Patricia DeMarrais, Camlin Tierney, Mary G Fowler, Lisa M Frenkel, Promoting Maternal and Infant Survival Everywhere (PROMISE) Study Team, Patricia M Flynn, Judith Currier, Susan Fiscus, Katherine Luzuriaga, Adriana Weinberg, James McIntyre, Tsungai Chipato, Lawrence Fox, Karin L Klingman, Renee Browning, Lynne M Mofenson, George K Siberry, Heather Watts, Lynette Purdue, David Shapiro, Terrence Fenton, Mae P Cababasay, Paula Britto, Yan Wang, Li Liu, Sean Brummel, Konstantia Angelidou, Michael Basar, Linda Millar, Kathleen Kaiser, John Gaeddert, Linda Marillo, Andrea Ciaranello, Kenneth Freedberg, Linda Barlow-Mosha, Mary Patricia Toye, Mark Mirochnick, Debika Bhattacharya, Amy Jennings, Adam Manzella, Amanda Zadzilka, William B Kabat, Amy James Loftis, Benjamin Chi, Marc Lallemant, Taha E Taha, Dhayendre Moodley, Karin Nielsen, Arlene Bardeguez, Anna Coutsoudis, Amita Gupta, Risa Hoffman, Elizabeth McFarland, Lynda Stranix-Chibanda, Gerhard B Theron, Lindiwe Msweli, Anne Coletti, Kathleen George, Megan Valentine, Marisol Martinez, James F Rooney, Oxana Ivanova, Danielle Poulin Porter, Wendy Snowden, Helen Watson, Harry Moultrie, Ashraf Coovadia, Renate Strehlau, Gerhard B Theron, Mark Cotton, Magdel Rossouw, Raziya Bobat, Motshidi Sebitloane, Dhayendre Moodley, Avy Violari, Portia Kamthunzi, Mina Hosseinipour, Newton Kumwenda, Mac Mallewa, Pendo Mlay, Anne Buchanan, Namwinga Chintu, Mwangelwa Mubiana-Mbewe, Maxensia Owor, Jim Aizire, Tsungai Chipato, Ramesh Bhosale, Sandhya Khadse

**Affiliations:** Department of Global Health, University of Washington, Seattle, Washington, USA; Center for Global Infectious Disease Research, Seattle Children's Research Institute, Seattle, Washington, USA; Center for Global Infectious Disease Research, Seattle Children's Research Institute, Seattle, Washington, USA; Center for Global Infectious Disease Research, Seattle Children's Research Institute, Seattle, Washington, USA; Center for Global Infectious Disease Research, Seattle Children's Research Institute, Seattle, Washington, USA; Center for Global Infectious Disease Research, Seattle Children's Research Institute, Seattle, Washington, USA; Center for Global Infectious Disease Research, Seattle Children's Research Institute, Seattle, Washington, USA; Center for Global Infectious Disease Research, Seattle Children's Research Institute, Seattle, Washington, USA; Center for Global Infectious Disease Research, Seattle Children's Research Institute, Seattle, Washington, USA; Center for Global Infectious Disease Research, Seattle Children's Research Institute, Seattle, Washington, USA; Center for Global Infectious Disease Research, Seattle Children's Research Institute, Seattle, Washington, USA; Center for Global Infectious Disease Research, Seattle Children's Research Institute, Seattle, Washington, USA; Center for Biostatistics in AIDS Research, Harvard T. H. Chan School of Public Health, Boston, Massachusetts, USA; Center for Biostatistics in AIDS Research, Harvard T. H. Chan School of Public Health, Boston, Massachusetts, USA; Department of Pathology, Johns Hopkins University, Baltimore, Maryland, USA; Department of Global Health, University of Washington, Seattle, Washington, USA; Center for Global Infectious Disease Research, Seattle Children's Research Institute, Seattle, Washington, USA; Department of Pediatrics, University of Washington, Seattle, Washington, USA; Department of Laboratory Medicine, University of Washington, Seattle, Washington, USA

**Keywords:** EFV, HIV, pretreatment drug resistance, TLE, virologic failure

## Abstract

**Background:**

Two large studies suggest that resistance mutations to only nonnucleoside reverse transcriptase inhibitors (NNRTI) did not increase the risk of virologic failure during antiretroviral therapy (ART) with efavirenz/tenofovir disoproxil fumarate/lamivudine (or emtricitabine). We retrospectively evaluated a third cohort to determine the impact of NNRTI resistance on the efficacy of efavirenz-based ART.

**Methods:**

Postpartum women living with human immunodeficiency virus (HIV) were studied if they initiated efavirenz-based ART because of the World Health Organization’s recommendation for universal ART. Resistance was detected by Sanger genotyping plasma prior to efavirenz-based ART and at virologic failure (HIV RNA >400 copies/mL). Logistic regression examined relationships between pre-efavirenz genotypes and virologic failure.

**Results:**

Pre-efavirenz resistance was detected in 169 of 1223 (13.8%) participants. By month 12 of efavirenz-based ART, 189 of 1233 (15.3%) participants had virologic failure. Rates of virologic failure did not differ by pre-efavirenz NNRTI resistance. However, while pre-efavirenz nucleos(t)ide reverse transcriptase inhibitors (NRTI) and NNRTI resistance was rare (8/1223 [0.7%]) this genotype increased the odds (adjusted odds ratio, 11.2 [95% confidence interval, 2.21–72.2]) of virologic failure during efavirenz-based ART. Age, time interval between last viremic visit and efavirenz initiation, clinical site, viremia at delivery, hepatitis B virus coinfection, and antepartum regimen were also associated with virologic failure.

**Conclusions:**

Resistance to NNRTI alone was prevalent and dual-class (NRTI and NNRTI) resistance was rare in this cohort, with only the latter associated with virologic failure. This confirms others’ findings that, if needed, efavirenz-based ART offers most people an effective alternative to dolutegravir-based ART.

Following the introduction of antiretroviral therapy (ART) in low- and middle-resource settings, multiple World Health Organization (WHO) surveillance studies detected pre-ART human immunodeficiency virus (HIV) drug resistance (PDR) prevalent at ≥10% [1]. The resistance mutations were primarily associated with resistance to nonnucleoside reverse transcriptase inhibitors (NNRTIs). This generated concern that PDR could undermine the efficacy of ART composed of tenofovir disoproxil fumarate (TDF)/lamivudine (3TC)/efavirenz (EFV) [2]. These concerns and the manufacturing of affordable TDF/3TC/dolutegravir (DTG) triggered the WHO in 2018 to recommend TDF/3TC/DTG for first-line ART [3]. However, prior to this recommendation, across 2006–2014 when PDR to NNRTIs was increasing, particularly among women of childbearing potential [4], and when first-line ART switched from zidovudine (ZDV)/3TC/nevirapine (NVP) to TDF/3TC/EFV, studies of Kenyans found that the impact of specific PDR mutational patterns on virologic failure appeared to vary depending on whether the NNRTI in the ART regimen was NVP or EFV [5, 6]. Specifically, K103N alone, which was the most frequent PDR mutational pattern, was associated with virologic failure to NVP/ZDV/3TC or NVP/stavudine/3TC but not to TDF/3TC/EFV [5]. Similarly, a study of South Africans (ANRS 12249) found that PDR to only NNRTIs (primarily K103N), including minority variants, had no detectable impact on viral suppression with predominantly TDF/emtricitabine (FTC)/EFV [7]. These findings are highly relevant, as both suggest that TDF/EFV combined with either 3TC or FTC (TXE) can be used effectively in people with K103N and perhaps other major NNRTI mutations—expanding available ART options. The present study assesses in an additional cohort the impact of PDR on virologic failure during EFV-based ART and evaluates specific mutant codons associated with virologic failure.

## MATERIALS AND METHODS

### Study Participants and Design

**Figure 1.**
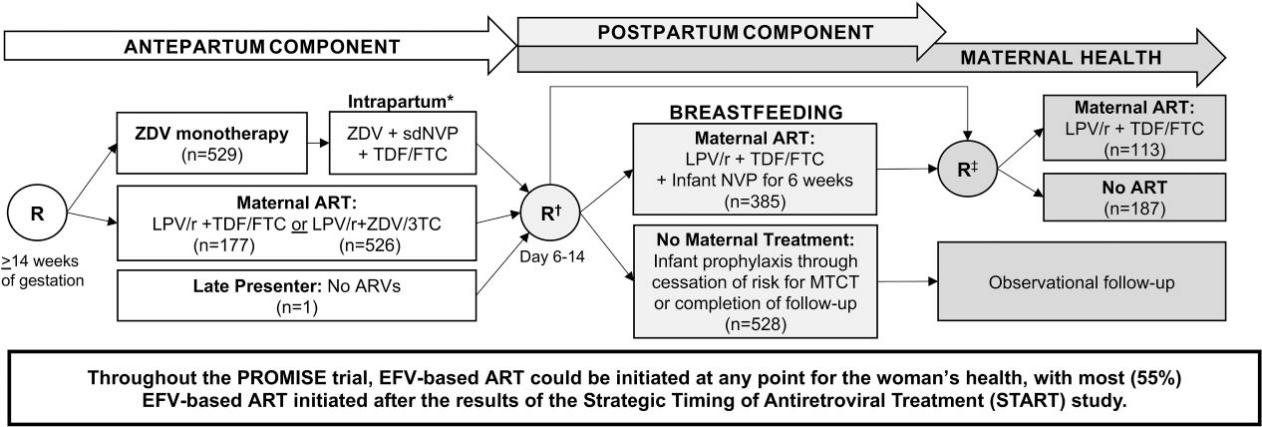
Promoting Maternal and Infant Survival Everywhere (PROMISE) trial randomization schema for efavirenz-based antiretroviral therapy (ART) substudy participants. The timing of all 3 antiretroviral drug randomizations are shown for each “component” of the PROMISE trial. The number of women in each PROMISE randomization group is shown in parentheses. Participants in this substudy cohort (N = 1233) had efavirenz-based ART prescribed (usually as tenofovir + lamivudine [or emtricitabine] + efavirenz) as part of local standard of care at any phase of the PROMISE trial. The impact of pre-efavirenz-based ART HIV drug resistance on ART suppression was assessed in women prescribed efavirenz-based ART at any phase of the PROMISE study. *Single-dose nevirapine was given at labor/delivery with a “tail” of tenofovir disoproxil fumarate /emtricitabine for 6–14 days to reduce the risk of resistance. †Eligible and willing antepartum and late-presenting mothers and their infants were randomized for the duration of breastfeeding; infants were to be followed to 104 weeks of age. Some mothers who were ineligible for the postpartum component were directly randomized to the maternal health component after delivery. ‡Randomization to the maternal health component occurred at breastfeeding cessation or at or after 74 weeks of breastfeeding for women randomized in the postpartum component. Those randomized directly to the maternal health component after delivery were randomized between 6 and 28 days postpartum. Participants randomized in the antepartum component and not randomized in the postpartum component remained in observational follow-up. Abbreviations: 3TC, lamivudine; ART, antiretroviral treatment; ARVs, antiretroviral drugs; EFV, efavirenz; FTC, emtricitabine; LPV/r, ritonavir-boosted lopinavir; MTCT, mother-to-child transmission; NVP, nevirapine; PROMISE, Promoting Maternal and Infant Survival Everywhere; R, randomization; sdNVP, single-dose nevirapine; TDF, tenofovir disoproxil fumarate; ZDV, zidovudine.

Women prescribed EFV-based ART during the Promoting Maternal and Infant Survival Everywhere (PROMISE) trial (see study schema in Figure 1) were retrospectively assessed for HIV drug resistance mutational patterns prior to EFV-based ART associated with virologic failure to this regimen [8]. PROMISE was a large, randomized trial that compared the relative safety and efficacy of 3 ART regimens for the prevention of mother-to-child HIV transmission during pregnancy in pregnant women living with HIV with CD4 counts above the level that (at the time) qualified for ART in sub-Saharan Africa and India. During the PROMISE trial, the TEMPRANO and strategic timing of antiretroviral therapy (START) trials demonstrated the benefits of starting ART despite “high” CD4 counts [9, 10]; thus, adult PROMISE participants could initiate EFV-based ART at any point for their health. Most PROMISE participants initiated EFV/TDF combined with 3TC (59%) or FTC (39%), or rarely (2%) combined with other nucleoside reverse transcriptase inhibitors (NRTIs) [11]. Therefore, EFV-based ART is also referred to as TXE in this study. In our study population, all participants initiated EFV-based ART during the postpartum or maternal health components of the PROMISE trial (Figure 1).

Inclusion criteria for this substudy were (1) initiation of EFV-based ART during PROMISE; (2) available plasma with HIV RNA >400 copies/mL prior to initiation of EFV-based ART; and (3) plasma HIV RNA measured at months 6 and 12 (±3 months) of EFV-based ART. Pre-EFV resistance—hereafter, PDR—and other parameters known to affect the likelihood of ART suppression were compared between participants who did versus did not have virologic failure (HIV RNA >400 copies/mL) by 12 months of EFV-based ART. PDR was assessed by Sanger sequences using participants’ viremic plasma nearest to the initiation of EFV-based ART. Genotypic resistance testing was also performed using first virologic failure plasma (month 6 or 12).

One covariate associated with virologic failure led to a post hoc study using next-generation sequencing of pre-EFV plasma. Plasma from cases and randomly selected controls (1:3) were tested to address the hypothesis that dual-class PDR with minority variants not detected by Sanger sequencing contributed to virologic failure.

### Laboratory Methods

RNA extracted from plasma (140–1000 μL) by QIAamp viral RNA mini kit (Qiagen, Valencia, California) was reverse transcribed, and HIV *pol* encoding protease and a portion of reverse transcriptase was amplified using PrimeScript One Step reverse-transcription polymerase chain reaction (PCR) (Takara Bio USA, Mountain View, California) followed by consensus Sanger sequencing [12]. For Illumina sequencing, RNA was reverse transcribed using SuperScript III First-Strand Synthesis System (Invitrogen, Carlsbad, California) and a primer consisting of an HIV-specific sequence (Supplementary Table 1) followed by a string of 8 random nucleotides (unique molecular identifier [UMI]) and a universal 24 bp Illumina index adapter sequence, then bead-purified (Agencourt Ampure XP, Beckman-Coulter, Beverly, Massachusetts). The complementary DNA was amplified (45 cycles; primers in Supplementary Table 1) using a high-fidelity PCR kit (FastStart High-Fidelity PCR system, Roche Diagnostics, Mannheim, Germany), then indexed (IDT for Illumina Nextera DNA Unique Dual Indexes, Illumina, San Diego, California) and pooled for bidirectional sequencing on an Illumina MiSeq (MiSeq Reagent Kit v3, Illumina).

### Bioinformatic Analyses

Sanger sequences (National Center for Biotechnology Information [NCBI] GenBank: OR390081–OR391479, MZ706694, MZ706718, MZ706834, MZ706873, and MZ706935) were analyzed by Sequencher (v5.4.6). Neighbor-joining phylogenetic trees of all sequences (Geneious v11.1.5) were utilized to detect potential specimen mix-ups or carry-over contamination between participants. Illumina sequences (NCBI Sequence Read Archive BioProject: PRJNA1000967) were processed [4], and ≥3 high-quality sequences with identical UMIs were combined into a consensus sequence (https://github.com/MullinsLab/drm-snp-calling) then aligned to the HXB2 using the Burrows-Wheeler algorithm [13] to identify nucleotides associated with resistance. The mutational frequencies are reported for participants with ≥100 consensus sequences using cut-offs of ≥20%, ≥5%, and ≥1% mutant.

PDR was defined by the 2009 WHO surveillance list of transmitted drug resistance mutations [14] for the primary analyses of the entire cohort and the post hoc case-control study. HIV drug resistance at virologic failure was defined by a genotypic susceptibility score (GSS) ≥10 for EFV, TDF, and/or XTC (Stanford HIVdb version 8.9–1) [15].

### Statistical Analyses

The prevalence of PDR at the initiation of EFV-based ART and at virologic failure was compared across clinical sites by χ^2^ test. Virologic failure between women with and without PDR was compared by Fisher exact test. Covariates associated with virologic failure were analyzed using unadjusted and adjusted logistic regression models of virologic failure during EFV-based ART, including PDR genotype, age, CD4 cell count, plasma HIV RNA, and detection of hepatitis B surface antigen (HBsAg) at PROMISE entry, HIV RNA load at EFV initiation, time (weeks) between delivery and EFV initiation, time (weeks) between last viremic visit and EFV initiation (pre-EFV specimen), time (weeks) on EFV, clinical site, delivery HIV RNA load, PROMISE antepartum randomization group, and PROMISE postpartum randomization group. To determine if mutations not defined as resistance mutations by Stanford may have contributed to virologic failure, we compared the frequency of mutations encoding reverse transcriptase “other” mutations between TDF-exposed versus TDF-unexposed participants prior to EFV-based ART and the rates of virologic failure among TDF-exposed participants with versus without each specific “other” mutations by Fisher exact test.

The post hoc case-control analyses of Sanger and Illumina sequences compared pre-EFV genotypes between women who experienced virologic failure versus suppression on EFV-based ART using unadjusted logistic regression. Comparisons were conducted using mutational frequencies of ≥20% mutant (assumed when detected by Sanger sequencing) and ≥5% and ≥1% mutant. To reduce bias and address data separation, Firth penalized likelihood approach was applied to logistic regression models [16, 17].

Sensitivity analyses were performed that defined PDR using mutations with GSS ≥10 by Stanford HIVdb to EFV, TDF, and/or XTC (instead of WHO resistance surveillance list). Participants with mutations not conferring resistance to TXE (eg, T215F or T215S conferring resistance to ZDV) were categorized as wild-type. All statistical analyses were conducted using RStudio (v4.2.1) with statistical significance defined as a 2-sided *P* value <.05 [18].

### Patient Consent Statement

This study addressed a secondary objective outlined in the trial protocol, which was approved by local and collaborating institutional review boards. All PROMISE trial participants provided written informed consent.

### Role of Funding Source

The funder had no role in study design; data generation, analysis, or interpretation; or manuscript writing.

## RESULTS

### Study Population

A total of 1233 PROMISE participants were studied. Demographic and clinical characteristics of participants prior to EFV initiation from 14 different sites in 7 countries are shown in Table 1. Participants were randomized to antepartum and postpartum regimens within the PROMISE protocol prior to EFV-based ART. The median time between delivery and EFV initiation was 112 weeks (interquartile range, 64–151 weeks).

**Table 1.** Baseline Demographic and Clinical Characteristics of Participants Prior to Initiation of Efavirenz-Based Antiretroviral Therapy

Characteristic	N = 1233
	Median (IQR) or no./No. (%)
Age, y	26 (22–30)
CD4 count at PROMISE entry, cells/μL	507 (428–632)
HIV RNA load at PROMISE entry, log_10_ copies/mL	3.92 (3.47–4.43)
HIV RNA load at EFV initiation, log_10_ copies/mL	3.87 (3.34–4.47)
Time between delivery and EFV initiation, wk	112 (64–151)
Time between last viremic visit and EFV initiation, wk	1.1 (0.0–24.1)
Time on EFV-based ART, wk	57.7 (42.3–82.1)
Country	
India	14 (1.1)
Malawi	353 (28.6)
South Africa	406 (32.9)
Tanzania	17 (1.4)
Uganda	82 (6.7)
Zambia	34 (2.8)
Zimbabwe	327 (26.5)
Clinical care site	
Site A	220 (17.8)
Site B	206 (16.7)
Site C	147 (11.9)
Site D	152 (12.3)
Site E	121 (9.8)
Site F	127 (10.3)
Sites G–N^a^	260 (21.1)
HIV RNA load at delivery^b^	
≤400 copies/mL	658 (53.4)
>400 copies/mL	542 (44.0)
Hepatitis B virus test result	
HBsAg negative	1191 (96.6)
HBsAg positive	42 (3.4)
PROMISE antepartum treatment regimen	
Triple ARV (ZDV/3TC + LPV/r)	526 (42.7)
Triple ARV (TDF/FTC + LPV/r)	177 (14.4)
ZDV monotherapy (+ sdNVP + TDF/FTC tail)	529 (42.9)
No antepartum randomization^c^	1
PROMISE postpartum treatment regimen	
Maternal triple ARV (TDF/FTC + LPV/r)	423 (34.3)
No maternal treatment	590 (47.9)
Not randomized (observational follow-up only)	220 (17.8)
HIV subtype^d^	
C	1128 (91.5)
A	59 (4.8)
D	27 (2.2)
B	7 (0.6)
G	2 (0.2)
HIV genotype at initiation of EFV-based ART^d^	
Wild-type	1054 (86.2)
Any resistance mutations	169 (13.8)
NRTI mutation(s) only	12 (1.0)
K103N only	97 (7.9)
Y181C only	12 (1.0)
G190A only	5 (0.4)
Other single NNRTI mutation	25 (2.0)
≥2 NNRTI mutations	10 (0.8)
≥1 NRTI and ≥1 NNRTI mutation	8 (0.7)

^a^Aggregated sites with <100 participants enrolled.

^b^Thirty-three participants did not have HIV RNA viral load measurements within 14 days of delivery.

^c^One participant was enrolled into PROMISE at delivery (ie, “late presenter”) and thus was not randomized during the antepartum component.

^d^Ten samples did not amplify and thus have no HIV subtype or genotype. Resistance to protease inhibitors was not detected in any participants.

Abbreviations: 3TC, lamivudine; ART, antiretroviral therapy; ARV, antiretroviral drug; EFV, efavirenz; FTC, emtricitabine; HBsAg, hepatitis B surface antigen; HIV, human immunodeficiency virus; IQR, interquartile range; LPV/r, ritonavir-boosted lopinavir; NNRTI, nonnucleoside reverse transcriptase inhibitor; NRTI, nucleos(t)ide reverse transcriptase inhibitor; PROMISE, Promoting Maternal and Infant Survival Everywhere; sdNVP, single-dose nevirapine; TDF, tenofovir disoproxil fumarate; ZDV, zidovudine.

### Prevalence of Pre-EFV-Based ART Drug Resistance

HIV genotypes were derived from pre-EFV plasma by Sanger sequencing for 1223 of 1233 (99.2%) participants (10 failed PCR amplification). Drug resistance was detected in 169 of 1223 (13.8% [95% confidence interval {CI}, 11.9%–15.9%]; Supplementary Figure 1*A*) with similar frequencies across sites (*P* = .8 by χ^2^ test). Among those with PDR, the most frequent mutation was K103N (97/169 [57.4%]) followed by other single NNRTI resistance mutations: L100I (n = 1), K101E (n = 15), K103S (n = 3), V106A/M (n = 3), Y181C (n = 7), Y188C (n = 1), G190A/E (n = 6), and P225H (n = 1). NRTI resistance mutations were infrequently detected (n = 12 [7.1%]), as were ≥2 NNRTI mutations (n = 10 [5.9%]), or dual-class resistance (≥1 NRTI and ≥1 NNRTI mutation) (n = 8 [4.7%]) (Table 1).

### Pre-EFV-Based ART Drug Resistance and Virologic Outcome During EFV-Based ART

Virologic failure was detected in 189 of 1233 women (15.3% [95% CI, 13.4%–17.5%]; Supplementary Figure 1*B*), observed by month 6 in 160 of 189 (84.7%) and by month 12 in 29 additional participants. Frequencies of virologic failure differed across clinical care sites (range, 4.6%–25.9%, *P* < .001 by χ^2^ test). PDR was not associated with virologic failure (PDR: 17.2% vs no PDR: 15.1%, *P* = .49; Supplementary Figure 2).

Dual-class resistance (defined as ≥1 NRTI and ≥1 NNRTI mutation) was the only PDR pattern associated with virologic failure during EFV-based ART in logistic regression models (Table 2) (unadjusted odds ratio [OR], 14.6 [95% CI, 3.70–80.0]; adjusted OR, 11.2 [95% CI, 2.21–72.2]). PDR with only NRTI mutation(s) or multiple NNRTI mutations were not associated with virologic failure compared to no PDR. Additionally, PDR with single major NNRTI mutations (K103N, Y181C, or G190A) was not associated with virologic failure during EFV-based ART. We observed that most participants with PDR due to a single pretreatment NNRTI mutation associated with high-level resistance to EFV (Stanford GSS ≥30) (n = 97/114 [85.1%]) had suppression of HIV replication during EFV-based ART (n = 81/97 [83.5%] with K103N, n = 10/12 [83.3%] with Y181C, and n = 5/5 [100%] with G190A). Comparison of pre-EFV genotypes across all PROMISE participants found that assignment to a TDF-containing regimen during PROMISE was associated with detection of V118I in the reverse transcriptase gene (6.2% vs 2.7%, *P* = .002, Supplementary Table 2); however, V118I among those randomized to TDF was not associated with virologic failure during EFV-based ART (4.6% vs 6.6%, *P* = .6).

**Table 2.** Comparison of Women With Virologic Failure Versus Antiretroviral Therapy (ART) Suppression During Efavirenz-Based ART

Characteristic	No.	Virologic Failure (n = 189)	ART-Suppressed (n = 1044)	Logistic Regression Model of Virologic Failure on EFV-Based ART	
		Median (IQR) or No. (%)		Unadjusted OR (95% CI)	Adjusted OR (95% CI)
Clinical data	1233				
Age, y (analyzed per 5 y)		24 (21–28)	26 (23–30)	0.66 (.56–.78)***	0.75 (.61–.90)**
CD4 count at PROMISE entry, cells/μL (analyzed per 50 cells/μL)		500 (431–623)	510 (426–632)	1.00 (.96–1.04)	1.01 (.96–1.06)
HIV RNA load at PROMISE entry, log_10_ copies/mL		4.03 (3.58–4.56)	3.90 (3.45–4.40)	1.33 (1.06–1.67)*	0.90 (.66–1.24)
HIV RNA load at EFV initiation, log_10_ copies/mL		3.95 (3.53–4.62)	3.85 (3.29–4.44)	1.32 (1.08–1.62)**	1.12 (.84–1.49)
Time between delivery and EFV initiation, wk (analyzed per 26 wk)		110 (66.0–145.0)	113 (63.8–153.0)	0.98 (.92–1.06)	0.96 (.86–1.06)
Time between last viremic^a^ visit and EFV initiation, wk (analyzed per 4 wk)		0.6 (0.0–9.1)	1.3 (0.0–26.4)	0.96 (.94–.98)***	0.96 (.93–.98)***
Time on EFV-based ART, wk		55.6 (36.4–78.9)	58.0 (43.3–82.6)	1.00 (.99–1.00)	1 (.99–1.00)
Clinical site^b^	1233				
Site A	220	25 (11.4)	195 (88.6)	Reference	Reference
Site B	206	48 (23.3)	158 (76.7)	2.35 (1.40–4.0)**	2.69 (1.51–4.92)***
Site C	147	38 (25.9)	109 (74.1)	2.70 (1.56–4.73)***	2.62 (1.39–5.01)**
Site D	152	7 (4.6)	145 (95.4)	0.40 (.16–.88)*	0.43 (.17–1.00)*
Site E	121	12 (9.9)	109 (90.1)	0.88 (.42–1.76)	1.25 (.52–2.83)
Site F	127	16 (12.6)	111 (87.4)	1.13 (.58–2.18)	1.23 (.58–2.53)
Sites G–N^c^	260	43 (16.5)	217 (83.5)	1.53 (.91–2.62)	1.53 (.86–2.80)
HIV RNA load at delivery	1200^d^				
≤400 copies/mL	658	72 (10.9)	586 (89.1)	Reference	Reference
>400 copies/mL	542	113 (20.8)	429 (79.2)	2.14 (1.56–2.95)***	2.71 (1.76–4.21)***
Hepatitis B virus test result	1233				
HBsAg negative	1191	188 (15.8)	1003 (84.2)	Reference	Reference
HBsAg positive	42	1 (2.4)	41 (97.6)	0.19 (.02–.72)*	0.17 (.02–.68)**
PROMISE antepartum treatment regimen	1232^e^				
Triple ARV (ZDV/3TC + LPV/r)	526	89 (16.9)	437 (83.1)	Reference	Reference
Triple ARV (TDF/FTC + LPV/r)	177	24 (13.6)	153 (86.4)	0.78 (.47–1.25)	0.69 (.38–1.21)
ZDV monotherapy (+ sdNVP + TDF/FTC tail)^f^	529	76 (14.4)	453 (85.6)	0.82 (.59–1.15)	0.48 (.31–.76)**
PROMISE postpartum treatment regimen	1013^g^				
Maternal triple ARV (TDF/FTC + LPV/r)	423	72 (17.0)	351 (83.0)	Reference	Reference
No maternal treatment	590	95 (16.1)	495 (83.9)	0.93 (.67–1.31)	0.68 (.46–1.00)
HIV genotype at EFV initiation^h^	1223^i^				
Wild-type	1054	159 (15.1)	895 (84.9)	Reference	Reference
Any resistance mutations	169	29 (17.2)	140 (82.8)	1.18 (.75–1.79)	1.08 (.65–1.75)
NRTI mutation(s) only	…	0	12	0.22 (.00–1.72)	0.13 (.00–1.19)
K103N only	…	16	81	1.14 (.63–1.93)	1.21 (.62–2.24)
Y181C only	…	2	10	1.34 (.26–4.67)	0.93 (.17–3.41)
G190A only	…	0	5	0.51 (.00–4.53)	0.40 (.00–4.40)
Other single NNRTI mutation	…	3	22	0.87 (.23–2.42)	0.84 (.21–2.60)
≥2 NNRTI mutations	…	2	8	1.65 (.31–6.04)	1.48 (.26–5.95)
≥1 NRTI and ≥1 NNRTI mutation	…	6	2	14.6 (3.70–80.0)***	11.2 (2.21–72.2)**

Unadjusted analyses examined the relationship with each covariate independently, whereas the adjusted logistic regression model included all covariates (age, CD4 count, HIV RNA load, interval between delivery and EFV initiation, interval between last viremic visit and EFV duration, duration of EFV, clinical site, hepatitis B test result, previous PROMISE treatment randomization arms, and genotype).

Abbreviations: 3TC, lamivudine; ART, antiretroviral therapy; ARV, antiretroviral drug; CI, confidence interval; EFV, efavirenz; FTC, emtricitabine; HBsAg, hepatitis B virus surface antigen; HIV, human immunodeficiency virus; IQR, interquartile range; LPV/r, ritonavir-boosted lopinavir; NNRTI, nonnucleoside reverse transcriptase inhibitor; NRTI, nucleos(t)ide reverse transcriptase inhibitor; OR, odds ratio; PROMISE, Promoting Maternal and Infant Survival Everywhere; sdNVP, single-dose nevirapine; TDF, tenofovir disoproxil fumarate; ZDV, zidovudine.

^a^Last viremic visit was the plasma specimen collected prior to and closest to EFV initiation with HIV RNA >400 copies/mL.

^b^Overall effect of clinical site on adjusted model of virologic failure was significant (*P* < .001), so pairwise comparisons were performed with clinical site A—which had the most participants enrolled—as the reference group.

^c^Sites with <100 participants enrolled were aggregated.

^d^Thirty-three participants did not have HIV RNA viral load measurements within 14 days of delivery: 4 who failed on EFV-based ART and 29 who suppressed on EFV-based ART.

^e^One participant enrolled after delivery and was not randomized in antepartum component of PROMISE.

^f^There was no difference in the odds of failing EFV-based ART when randomized to the ZDV monotherapy arm (reference group) versus the triple ARV (TDF/FTC + LPV/r) arm during the antepartum component (OR, 0.94 [95% CI, .57–1.53]; *P* = .9). Results of Fisher exact test shown in Supplementary Table 3.

^g^Comparison only included the women who were randomized after delivery in PROMISE, either to the postpartum component or directly to the maternal health component, to evaluate the effect of triple ARV versus no treatment on virologic failure on EFV-based ART. Women who were not randomized after delivery (observational follow-up only) were excluded from this analysis (n = 220) as this group did not have a uniform treatment assignment.

^h^Two separate models (both including all other covariates in the table) were generated to examine the effects of genotype; one to compare any drug resistance detected vs wild-type and the other to compare each mutational category (n = 7) to wild-type.

^i^Ten samples did not amplify and thus have no genotype.

^*^ *P* < .05, **P < .01, ***P < .001 by logistic regression with Firth bias reduction.

Additional covariates associated with virologic failure during EFV-based ART in the adjusted logistic regression include enrollment at clinical site B (adjusted OR compared to site A, 2.69 [95% CI, 1.51–4.92]) and site C (adjusted OR, 2.62 [95% CI, 1.39–5.01]), previous virologic failure at delivery (adjusted OR, 2.71 [95% CI, 1.76–4.21]), younger age (*P* < .01), shorter time interval between last viremic study visit (pre-EFV genotype) and EFV initiation (*P* < .001), detection of HBsAg (*P* < .01), and prior PROMISE antepartum treatment randomization (*P* < .001) (Table 2, Supplementary Table 3). CD4 count at entry into the PROMISE trial, the time interval between delivery and EFV initiation, the duration of EFV-based ART, and the PROMISE postpartum treatment randomization were not associated with virologic failure on EFV-based ART.

Our finding that the PDR genotypes associated with virologic failure during EFV-based ART varied by prior PROMISE antepartum treatment arm (Table 3) in the adjusted model prompted additional comparisons. Among the majority (86%) of PROMISE participants randomized to antepartum ZDV monotherapy or ZDV/3TC + lopinavir/ritonavir (LPV/r), only dual-class PDR was associated with virologic failure during EFV-based ART (OR, 9.43 [95% CI, 1.24–104] and OR, 14.6 [95% CI, 2.66–148], respectively). In contrast, among 177 (14.4%) of PROMISE participants randomized to antepartum TDF/FTC + LPV/r, any PDR mutation was associated with virologic failure (OR, 4.21 [95% CI, 1.55–11.1]), including single major NNRTI mutations K103N and Y181C (OR, 5.02 [95% CI 1.47–16.0] and OR, 8.68 [95% CI, 1.26–60.1], respectively). An interaction test (*P* = .018) found that the genotype effect in the adjusted virologic failure model depended on the prior antepartum treatment randomization arm. Among participants with virologic failure, a greater proportion had PDR in the TDF/FTC + LPV/r arm compared to the other 2 antepartum arms (34.8% vs 10.5% for ZDV monotherapy vs 14.6% for ZDV/3TC + LPV/r; Table 3). However, the proportion with NNRTI PDR was not significantly greater in participants randomized to TDF/FTC + LPV/r compared to the other 2 regimens (14.4% [95% CI, 9.5%–20.5%] vs 11.8% [95% CI, 9.2%–14.9%] for ZDV monotherapy or 13.4% [95% CI, 10.6%–16.6%] for ZDV/3TC + LPV/r).

**Table 3.** Comparison of Women With Virologic Failure Versus Antiretroviral Therapy (ART) Suppression During Efavirenz-Based ART Within Prior PROMISE Antepartum Treatment Arms

Characteristic	Antepartum ZDV Monotherapy			Antepartum ZDV/3TC + LPV/r			Antepartum TDF/FTC + LPV/r		
	Virologic Failure (n = 76)	ART-Suppressed (n = 453)	Unadjusted OR (95% CI)	Virologic Failure (n = 89)	ART-Suppressed (n = 437)	Unadjusted OR (95% CI)	Virologic Failure (n = 24)	ART-Suppressed (n = 153)	Unadjusted OR (95% CI)
Clinical data, median (IQR)									
Age, y (analyzed per 5 y)	24 (21–27)	26 (23–30)	0.65 (0.49–0.84)**	24 (21–28)	27 (23–31)	0.67 (0.53–0.85)***	22.5 (20.8–28)	26 (23–30)	0.67 (0.41–1.05)
HIV RNA load at PROMISE entry, log_10_ copies/mL	3.95 (3.56–4.56)	3.88 (3.47–4.35)	1.30 (0.90–1.87)	4.12 (3.70–4.60)	3.91 (3.42–4.45)	1.44 (1.03–2.00)*	4.24 (3.54–4.44)	3.99 (3.57–4.42)	1.09 (0.58–2.00)
HIV RNA load at EFV initiation, log_10_ copies/mL	4.02 (3.64–4.74)	3.97 (3.30–4.51)	1.43 (1.06–1.94)*	3.82 (3.46–4.57)	3.74 (3.28–4.37)	1.23 (0.90–1.69)	3.96 (3.51–4.54)	3.91 (3.36–4.45)	1.39 (0.77–2.53)
Time between delivery and EFV initiation, wk (analyzed per 26 wk)	4.22 (2.73–5.66)	4.73 (2.85–6.15)	0.94 (0.84–1.05)	4.77 (2.54–5.92)	4.69 (2.96–6.23)	0.97 (0.87–1.08)	2.88 (2.41–4.01)	2.42 (1.46–3.35)	1.25 (0.96–1.63)
Time between last viremic visit and EFV initiation, wk (analyzed per 4 wk)	0.04 (0–0.94)	0.29 (0–6.21)	0.96 (0.92–0.99)**	0.21 (0–3.11)	0.29 (0–7.29)	0.97 (0.95–1.00)*	0.25 (0–3.54)	0.71 (0–6.18)	0.96 (0.88–1.02)
HIV RNA load at delivery									
≤400 copies/mL	12 (15.8)	107 (23.6)	Reference	49 (55.1)	364 (83.3)	Reference	11 (45.8)	115 (75.2)	Reference
>400 copies/mL	62 (81.6)	324 (71.5)	1.66 (.90–3.29)	39 (43.8)	68 (15.6)	4.25 (2.59–6.95)***	12 (50)	36 (23.5)	3.44 (1.42–8.44)**
Hepatitis B virus test result									
HBsAg negative	76 (100)	439 (96.9)	Reference	89 (100)	425 (97.3)	Reference	23 (95.8)	138 (90.2)	Reference
HBsAg positive	0 (0)	14 (3.1)	0.2 (.00–1.51)	0 (0)	12 (2.8)	0.19 (.00–1.47)	1 (4.17)	15 (9.80)	0.57 (.06–2.48)
HIV genotype at EFV initiation^a,b^									
Wild-type	68 (89.5)	387 (86.4)	Reference	76 (85.4)	373 (85.8)	Reference	15 (65.2)	134 (88.7)	Reference
Any resistance mutations	8 (10.5)	61 (13.6)	0.78 (.34–1.60)	13 (14.6)	62 (14.3)	1.05 (.54–1.94)	8 (34.8)	17 (11.3)	4.21 (1.55–11.1)**
NRTI mutation(s) only	0	7	0.38 (.00–3.16)	0	5	0.44 (.00–3.98)	0	0	…
K103N only	5	38	0.81 (.29–1.90)	6	34	0.92 (.35–2.07)	5	9	5.02 (1.47–16.0)*
Y181C only	0	3	0.81 (.01–8.47)	0	5	0.44 (.00–3.98)	2	2	8.68 (1.26–60.1)*
G190A only	0	2	1.13 (.01–14.1)	0	1	1.63 (.01–30.8)	0	2	1.74 (.01–22.6)
Other single NNRTI mutation	0	7	0.38 (.00–3.16)	2	11	1.06 (.20–3.69)	1	4	2.89 (.28–17.0)
≥2 NNRTI mutations	1	3	2.42 (.23–15.0)	1	5	1.33 (.14–6.80)	0	0	…
≥1 NRTI and ≥1 NNRTI mutation	2	1	9.43 (1.24–104)*	4	1	14.6 (2.66–148)**	0	0	…

Abbreviations: 3TC, lamivudine; ART, antiretroviral therapy; CI, confidence interval; EFV, efavirenz; FTC, emtricitabine; HBsAg, hepatitis B virus surface antigen; HIV, human immunodeficiency virus; IQR, interquartile range; LPV/r, ritonavir-boosted lopinavir; NNRTI, nonnucleoside reverse transcriptase inhibitor; NRTI, nucleos(t)ide reverse transcriptase inhibitor; OR, odds ratio; TDF, tenofovir disoproxil fumarate; ZDV, zidovudine.

^a^Two separate models were generated to examine the effects of genotype; one to compare any drug resistance detected vs wild-type and the other to compare each mutational category (n = 7) to wild-type.

^b^Samples without genotype (n = 10) did not amplify.

^*^ *P* < .05, **P < .01, ***P < .001 by unadjusted logistic regression with Firth bias reduction.

Illumina sequences from 22 of 24 cases and 79 of 80 controls evaluated whether PDR patterns in the TDF/FTC + LPV/r arm including low-frequency TDF or FTC resistance mutations missed by Sanger genotyping were associated with virologic failure during EFV-based ART. A comparison of PDR by Sanger and Illumina sequences, using a threshold frequency of 20%, found concordant genotypes for 100 of 101 (99.0%) participants based on the WHO surveillance mutations list [14]; the discordant result was in a control with K103N detected at a frequency of 25% by Illumina that was not detected by Sanger sequencing. In our sensitivity analyses defining PDR by Stanford's GSS ≥10 to EFV, TDF, and/or XTC, we detected 3 discordant results: K70N in 1 participant and K238T in another participant were missed by Illumina sequencing, and A98G detected at 26.2% frequency by Illumina was not detected by Sanger. While the Stanford HIVdb gives a GSS ≥10 to EFV, TDF, and/or XTC for these 3 mutations, these codons are not included in the WHO list of surveillance mutations. Additionally, while Illumina detected resistance mutations at <20% frequencies (n = 20/101 and n = 25/101 using 5% and 1% thresholds, respectively), none of these mutations conferred resistance to TDF (Supplementary Table 4). NRTI resistance was detected at <20% frequency in 0 of 22 cases and 2 of 79 (2.5%) controls: M184I at 1.8% in 1 participant alone and D67G at 2.2% in another participant who also had K101E at 4.2%. The inclusion of minority variants recategorized, at most, 2 case and 7 control participants’ genotypes, primarily from single to multiple NNRTI mutations (no additional participants with dual NRTI + NNRTI resistance mutations); thus, the increased odds of virologic failure associated with NNRTI PDR was maintained (Supplementary Tables 4 and 5).

Sensitivity analyses repeated the pairwise comparisons of pre-EFV genotypes associated with virologic failure using Stanford HIVdb GSS ≥10 to EFV/TDF/XTC and yielded results similar to primary analyses (Supplementary Tables 5–7). The minor differences were in the subgroup analyses of the antepartum TDF/FTC + LPV/r arm with single NNRTI mutations, ≥2 NNRTI mutations, and dual-class PDR associated with virologic failure in the unadjusted model (Supplementary Table 7).

### HIV Drug Resistance at Virologic Failure

Of the 189 women with virologic failure during EFV-based ART, 180 (95.2%) genotypes were derived from their first failure timepoint (month 6 or month 12) and 9 specimens failed PCR amplification. Sixty-three of 159 (44.8%) women without PDR had drug-resistant variants detected at virologic failure. However, more than half (89/159 [55.2%]) had wild-type HIV at virologic failure during EFV-based ART (Table 4). In contrast, nearly all women with PDR maintained detectable resistance (25/29 [89.7%]) at virologic failure (OR, 11.6 [95% CI, 3.33–62.8]; *P* < .0001 by Fisher exact test). Wild-type HIV at failure was detected in 3 of 29 (10.3%) with PDR, with V106VA and Y181YC no longer detected in 1 woman and K103KN in another 2 women. Among participants randomized in PROMISE to antepartum TDF/FTC + LPV/r, no new NRTI resistance mutations were detected in any of the 23 of 24 with virologic failure during EFV-based ART successfully genotyped; the 10 who had NNRTI PDR maintained these variants at virologic failure, with 3 accumulating additional NNRTI mutations and 4 with detection of emergent NNRTI mutations.

**Table 4.** Prevalence of Human Immunodeficiency Virus Drug Resistance Among Participants With Virologic Failure During Efavirenz-Based Antiretroviral Therapy

HIV Genotype Prior to Initiation of EFV-Based ART (n = 189)^a^	No. (%) Wild-type at Virologic Failure	No. (%) Drug Resistant at Virologic Failure	Odds Ratio (95% CI)
Wild-type (n = 159)^b^	89 (55.2)	63 (44.8)	Reference
Drug-resistant (n = 29)^c^	3 (10.3)	25 (89.7)	11.6 (3.33–62.8)***

Abbreviations: ART, antiretroviral therapy; CI, confidence interval; EFV, efavirenz; HIV, human immunodeficiency virus.

^a^One (0.9%) specimen was not sequenced prior to EFV-based ART initiation.

^b^Seven (5.0%) specimens were not sequenced at virologic failure.

^c^One (3.0%) specimen was not sequenced at virologic failure.

^***^ *P* < .0001 by Fisher exact test.

## DISCUSSION

Salient observations from this study include that NNRTI resistance mutations were prevalent among women prior to EFV-based ART initiation; however, NNRTI resistance mutations alone were not associated with virologic failure to EFV-based ART, except in a subset of the cohort randomized antepartum to TDF/FTC + LPV/r. Detection of NRTI + NNRTI resistance mutations were infrequent in this cohort; however, this dual-class drug resistance was strongly associated with virologic failure. Among women with virologic failure during EFV-based ART, testing at the time of virologic failure found that more than half maintained their wild-type genotypes.

It is notable that most study participants with PDR comprised of major NNRTI mutations, including K103N (estimated to confer a 15- to 40-fold reduction in EFV susceptibility for subtype C [19]), had suppression of HIV replication during treatment with TXE. This finding is consistent with studies of Kenyans and South Africans in whom PDR to NNRTI alone did not affect virologic outcome to EFV-based ART [5, 7]. Together, these data suggest that the combination of TXE, presumably with good adherence, is sufficiently potent to suppress viral replication despite the presence of NNRTI PDR. A systematic review of the virological efficacy of 4 regimens, including TDF/3TC/NVP, TDF/FTC/NVP, TDF/3TC/EFV, and TDF/FTC/EFV, found EFV/TDF/FTC to have equivalent or superior efficacy to its comparator arms across 4 comparator studies, possibly due to the higher potency of EFV compared to NVP, with potential contributions by FTC due to its longer intracellular half-life compared to 3TC [20]. The greater activity of EFV at achievable blood levels compared to NVP may explain our observation that NNRTI PDR did not diminish overall efficacy of EFV-based ART. Additionally, a slower metabolism of EFV due to cytochrome p450 CYP2B6 polymorphisms prevalent among Africans may have further increased the potency of EFV [21, 22] and contributed to EFV-based ART suppression of HIV replication despite NNRTI PDR.

The observation that any PDR was associated with virologic failure during EFV-based ART in the subanalyses of participants assigned to antepartum TDF/FTC + LPV/r in PROMISE differed from the larger group of participants. Our hypothesis that prior TDF/FTC exposure selected resistant variants with diminished replication capacity that persisted as minority variants was not supported by our Illumina studies. Whether minority variants persisted on the same viral template as NNRTI resistance mutations at frequencies below our limit of detection is unlikely, given that no additional NRTI mutations were detected among these participants at virologic failure. However, this subgroup differed from the other antepartum arms by later enrollment into PROMISE and thus a shorter interval between their antepartum TDF/FTC + LPV/r and EFV-based ART. We speculate that these mothers may have been administered routine but unrecorded single-dose NVP in labor, and that mutations selected by NVP may have contributed to virologic failure. Studies evaluating the effects of single-dose NVP on later NVP-based ART have found that a shorter interval between single-dose NVP and later ART is associated with virologic failure [23, 24], most likely because replication-competent HIV variants “decay” with time [25, 26].

In our overall adjusted model, clinical site, age, interval between last viremic visit and EFV initiation, and virologic status at prior delivery were all associated with virologic outcome on EFV-based ART. The association of clinical site may be attributed to geographical or site-specific characteristics that could contribute to nonadherence to EFV-based ART resulting in virologic failure, which has been reported previously [27]. Age could be an indicator of a more mature approach to treatment adherence and is consistent with prior studies associating younger age with lower rates of ART adherence [28, 29]. Previous virologic failure at delivery during PROMISE and shorter interval between last viremic study visit and EFV initiation could indicate a pattern of nonadherence to treatment. Others have shown that historical nonadherence is predictive of future treatment failure [30, 31]. The large proportion of women (48.5%) whose virologic failure genotype was wild-type, which was variable across sites, provides additional evidence of potential nonadherence; however, our analyses were limited by lack of objective adherence measurements.

There were several additional limitations to this study. The PROMISE trial inclusion criteria limited the study population to pregnant women with HIV who, due to a lack of disease and high CD4 counts, did not qualify for ART, and most participants (92.4%) we studied had viral load <5 log copies/mL at PROMISE enrollment. Therefore, our findings may not be representative of all women with HIV, particularly women with low CD4 cell counts due to HIV disease progression. The variation in virologic failure rates across clinical sites suggests that barriers to drug adherence differed between sites, particularly at the sites where virologic failure with wild-type HIV was more common. This may result in an underestimation of the association between PDR and virologic failure by month 6 or 12 of treatment; however, it is notable that the lack of an association between NNRTI PDR and treatment outcome in the previously published studies were in cohorts with relatively low rates of virologic failure (Kenyans [5.8%] and South Africans [5.5%]) [5, 7]. While we evaluated minority variants in a subset of women randomized to antepartum TDF/FTC + LPV/r within PROMISE, low-frequency drug resistance variants were not examined in all study participants, so it is uncertain if low-frequency mutations impacted treatment outcomes in others; however, minority variants did not appear relevant in either the Kenyan or South African cohorts [5, 7]. Additionally, the discordance between Illumina and Sanger genotypes detected in 3 participants suggests that primer bias, rather than sampling depth, affected our Sanger and/or next-generation sequencing, as >300 templates were sequenced by Illumina for all 3 discordant specimens. The post hoc comparison was comprised of a relatively small population (∼14% of the cohort) and the validity of these results is uncertain given the zero and single-digit event numbers that give low precision ORs. Last, analyses were focused on participants who completed at least 1 year of follow-up (and had specimens available), which limits the generalizability of our findings to participants with ≥12 months of follow-up on EFV-based ART.

In summary, this study found that the most prevalent NNRTI HIV drug resistance mutation, K103N, does not appear to increase the odds of virologic failure to EFV-based ART, except potentially in individuals previously administered TDF. The latter exception was from post hoc analysis of a relatively small population, which has not been confirmed in other studies. Dual class NNRTI + NRTI PDR was strongly associated with virologic failure during EFV-based ART in this study. However, the prevalence of dual class PDR was low in our study, which suggests that pretreatment genotypic testing prior to first-line ART initiation may not improve treatment outcomes for most people, which is consistent with current WHO guidelines. A potential exception is individuals infected during preexposure prophylaxis, although notably the prevalence of transmitted NRTI resistance has remained low, particularly for TDF-associated mutations [1]. In conclusion, our data, along with those from 2 other studies [5, 7], suggest that despite a high prevalence of NNRTI resistance in resource-limited settings, TXE could offer an effective HIV treatment for most individuals living with HIV. While TDF/3TC/DTG is currently recommended by the WHO for first- and second-line ART, given the limited number of affordable antiretrovirals in resource-limited settings, TXE should be regarded as a potential alternative regimen when TDF/3TC/DTG is contraindicated or not well tolerated.

## Supplementary Data

Supplementary materials are available at *Open Forum Infectious Diseases* online. Consisting of data provided by the authors to benefit the reader, the posted materials are not copyedited and are the sole responsibility of the authors, so questions or comments should be addressed to the corresponding author.

## Supplementary Material

ofae383_Supplementary_Data

## Notes

***Acknowledgments.*** The authors acknowledge the contributions of the study participants, site investigators and staff, and International Maternal Pediatric Adolescent AIDS Clinical Trials Network (IMPAACT) central resources who supported the 1077BF study.

***Prior presentation.*** 27th International Workshop on HIV Drug Resistance and Treatment Strategies, Johannesburg, South Africa, 22–24 October 2018.

***Data sharing.*** Nucleotide consensus sequences generated by Sanger sequencing are available in the NCBI GenBank (accession numbers OR390081-OR391479, MZ706694, MZ706718, MZ706834, MZ706873, and MZ706935), and Illumina data are available as raw sequencing reads in the NCBI Sequence Read Archive under BioProject number PRJNA1000967.

***Financial support*.** Support for this analysis and overall support for IMPAACT was provided by the National Institute of Allergy and Infectious Diseases (NIAID) with co-funding from the Eunice Kennedy Shriver National Institute of Child Health and Human Development (NICHD) and the National Institute of Mental Health, all components of the National Institutes of Health (grant numbers UM1AI068632 [IMPAACT Leadership and Operations Center], UM1AI068616 [IMPAACT Statistical and Data Management Center], and UM1AI106716 [IMPAACT Laboratory Center]) and by the NICHD (HHSN275201800001I). Additional support came from the Molecular Profiling and Computational Biology Core of the University of Washington Fred Hutch Center for AIDS Research (P30 AI027757 to L. M. F.), NICHD (HHSN275201300003C to L. M. F.), and NIAID (T32 AI007509 to C. L. B.). The study products were provided free of charge by Abbott, Gilead Sciences, Boehringer Ingelheim, and GlaxoSmithKline.
