# Supplementary material for: Impact of Human Immunodeficiency Virus Drug Resistance Mutations Detected in Women Prior to Antiretroviral Therapy With Efavirenz + Tenofovir Disoproxil Fumarate + Lamivudine (or Emtricitabine)
Source: Open Forum Infect Dis. 2024 Jul 15;11(7):ofae383. doi: 10.1093/ofid/ofae383 (PMC11267229; doi:10.1093/ofid/ofae383)
Supplement: ofae383_Supplementary_Data [file ofae383_supplementary_data.docx]

**SUPPLEMENTARY MATERIAL**

**Supplementary Table 1. Sequences of primers used for Illumina library preparation of samples in case-control study**

| Primer | Primer Sequences^a^  (5’ to 3’) | HXB2 Location |
| --- | --- | --- |
| cDNA | **CTCGGAGATGTGTATAAGAGACAG**NNNNNNNNCTGKACTGTCCAYTTGTCAGGATG | 3252←3275 |
| Forward PCR | **TCGTCGGCAGCGTCAGATGTGTATAAGAGACAG**AAACAATGGCCATTRACAGAAGA | 2613→2635 |
| Reverse PCR | **GTCTCGTGGGCTCGGAGATGTGTATAAGAGACAG** | N/A |

^a^ Bold font indicates Illumina adapter sequence; HIV-specific portion of primer is denoted by regular font

**Supplementary Figure 1: Prevalence of pretreatment drug resistance to nucleoside/tide or non-nucleoside reverse transcriptase inhibitors before efavirenz-based-ART initiation and at virologic failure of efavirenz-based-ART across clinical sites and overall.** Clinical sites with fewer than 100 participants were aggregated (sites G through N) due to small sample groups ranging between 4-82 participants. Error bars indicate 95% confidence interval. A) Proportion of women with pretreatment drug resistance across clinical sites and overall. Note: 10 out of 1233 specimens were not successfully sequenced due to no amplification. B) Proportion of women with virologic failure (HIV RNA >400 copies/mL) across sites and overall.

**Supplementary Figure 2: Proportion with virologic failure plotted by pre-efavirenz-based-ART genotype across clinical sites and overall.** Error bars indicate 95% confidence intervals. Note: 10 (0.8%) specimens failed PCR-amplification and therefore were not sequenced.

**Supplementary Table 2. Comparison of “other” mutations detected in reverse transcriptase gene between participants with vs. without prior tenofovir exposure before efavirenz-based-ART initiation**

| RT Codon | No Prior TDF, N = 678  n (%) | Prior TDF, N = 545  n (%) | p-value |
| --- | --- | --- | --- |
| P1 | 11 (1.6%) | 11 (2.0%) | 0.6 |
| S3 | 2 (0.3%) | 0 (0%) | 0.5 |
| P4 | 83 (12%) | 60 (11%) | 0.5 |
| I5 | 11 (1.6%) | 11 (2.0%) | 0.6 |
| E6 | 76 (11%) | 66 (12%) | 0.6 |
| T7 | 5 (0.7%) | 4 (0.7%) | >0.9 |
| V8 | 23 (3.4%) | 14 (2.6%) | 0.4 |
| P9 | 0 (0%) | 2 (0.4%) | 0.2 |
| V10 | 1 (0.1%) | 1 (0.2%) | >0.9 |
| K11 | 49 (7.2%) | 25 (4.6%) | 0.05 |
| K13 | 0 (0%) | 2 (0.4%) | 0.2 |
| P14 | 4 (0.6%) | 3 (0.6%) | >0.9 |
| G15 | 2 (0.3%) | 0 (0%) | 0.5 |
| M16 | 2 (0.3%) | 0 (0%) | 0.5 |
| D17 | 527 (78%) | 431 (79%) | 0.6 |
| P19 | 0 (0%) | 1 (0.2%) | 0.4 |
| K20 | 102 (15%) | 79 (14%) | 0.8 |
| V21 | 35 (5.2%) | 40 (7.3%) | 0.1 |
| K22 | 5 (0.7%) | 4 (0.7%) | >0.9 |
| Q23 | 0 (0%) | 1 (0.2%) | 0.4 |
| L26 | 1 (0.1%) | 0 (0%) | >0.9 |
| T27 | 26 (3.8%) | 32 (5.9%) | 0.1 |
| E28 | 17 (2.5%) | 12 (2.2%) | 0.7 |
| E29 | 2 (0.3%) | 1 (0.2%) | >0.9 |
| I31 | 2 (0.3%) | 3 (0.6%) | 0.7 |
| K32 | 41 (6.0%) | 25 (4.6%) | 0.3 |
| V35 | 676 (100%) | 540 (99%) | 0.3 |
| E36 | 470 (69%) | 373 (68%) | 0.7 |
| I37 | 1 (0.1%) | 0 (0%) | >0.9 |
| T39 | 637 (94%) | 516 (95%) | 0.6 |
| E40 | 74 (11%) | 50 (9.2%) | 0.3 |
| M41 | 1 (0.1%) | 0 (0%) | >0.9 |
| E42 | 2 (0.3%) | 1 (0.2%) | >0.9 |
| K43 | 25 (3.7%) | 19 (3.5%) | 0.9 |
| G45 | 1 (0.1%) | 0 (0%) | >0.9 |
| K46 | 0 (0%) | 1 (0.2%) | 0.4 |
| I47 | 2 (0.3%) | 1 (0.2%) | >0.9 |
| S48 | 592 (87%) | 481 (88%) | 0.6 |
| K49 | 58 (8.6%) | 35 (6.4%) | 0.2 |
| I50 | 7 (1.0%) | 4 (0.7%) | 0.8 |
| E53 | 19 (2.8%) | 19 (3.5%) | 0.5 |
| N54 | 1 (0.1%) | 0 (0%) | >0.9 |
| P55 | 1 (0.1%) | 0 (0%) | >0.9 |
| N57 | 0 (0%) | 1 (0.2%) | 0.4 |
| V60 | 256 (38%) | 204 (37%) | >0.9 |
| A62 | 1 (0.1%) | 0 (0%) | >0.9 |
| K64 | 12 (1.8%) | 13 (2.4%) | 0.4 |
| T69 | 6 (0.9%) | 1 (0.2%) | 0.1 |
| R72 | 1 (0.1%) | 0 (0%) | >0.9 |
| D76 | 1 (0.1%) | 1 (0.2%) | >0.9 |
| R78 | 0 (0%) | 1 (0.2%) | 0.4 |
| E79 | 1 (0.1%) | 3 (0.6%) | 0.3 |
| K82 | 3 (0.4%) | 7 (1.3%) | 0.1 |
| R83 | 1 (0.1%) | 1 (0.2%) | >0.9 |
| T84 | 1 (0.1%) | 0 (0%) | >0.9 |
| D86 | 4 (0.6%) | 3 (0.6%) | >0.9 |
| F87 | 1 (0.1%) | 0 (0%) | >0.9 |
| W88 | 18 (2.7%) | 6 (1.1%) | 0.05 |
| E89 | 1 (0.1%) | 0 (0%) | >0.9 |
| V90 | 12 (1.8%) | 11 (2.0%) | 0.8 |
| I94 | 0 (0%) | 1 (0.2%) | 0.4 |
| A98 | 15 (2.2%) | 19 (3.5%) | 0.2 |
| G99 | 0 (0%) | 1 (0.2%) | 0.4 |
| K101 | 8 (1.2%) | 8 (1.5%) | 0.7 |
| K102 | 29 (4.3%) | 22 (4.0%) | 0.8 |
| K103 | 11 (1.6%) | 6 (1.1%) | 0.4 |
| K104 | 18 (2.7%) | 25 (4.6%) | 0.07 |
| S105 | 1 (0.1%) | 0 (0%) | >0.9 |
| T107 | 2 (0.3%) | 1 (0.2%) | >0.9 |
| D110 | 1 (0.1%) | 0 (0%) | >0.9 |
| V111 | 1 (0.1%) | 0 (0%) | >0.9 |
| D113 | 3 (0.4%) | 0 (0%) | 0.3 |
| V118 | 18 (2.7%) | 34 (6.2%) | **0.002**** |
| P119 | 1 (0.1%) | 0 (0%) | >0.9 |
| D121 | 79 (12%) | 47 (8.6%) | 0.08 |
| K122 | 611 (90%) | 470 (86%) | **0.035*** |
| D123 | 481 (71%) | 360 (66%) | 0.07 |
| R125 | 4 (0.6%) | 2 (0.4%) | 0.7 |
| K126 | 1 (0.1%) | 1 (0.2%) | >0.9 |
| T131 | 2 (0.3%) | 1 (0.2%) | >0.9 |
| P133 | 1 (0.1%) | 1 (0.2%) | >0.9 |
| S134 | 0 (0%) | 1 (0.2%) | 0.4 |
| I135 | 157 (23%) | 137 (25%) | 0.4 |
| N137 | 0 (0%) | 1 (0.2%) | 0.4 |
| E138 | 2 (0.3%) | 1 (0.2%) | >0.9 |
| T139 | 28 (4.1%) | 17 (3.1%) | 0.4 |
| I142 | 51 (7.5%) | 47 (8.6%) | 0.5 |
| R143 | 1 (0.1%) | 0 (0%) | >0.9 |
| Y144 | 1 (0.1%) | 1 (0.2%) | >0.9 |
| Q145 | 5 (0.7%) | 11 (2.0%) | 0.05 |
| Y146 | 0 (0%) | 1 (0.2%) | 0.4 |
| G152 | 1 (0.1%) | 0 (0%) | >0.9 |
| W153 | 1 (0.1%) | 1 (0.2%) | >0.9 |
| A158 | 62 (9.1%) | 48 (8.8%) | 0.8 |
| I159 | 5 (0.7%) | 4 (0.7%) | >0.9 |
| S162 | 170 (25%) | 137 (25%) | >0.9 |
| S163 | 7 (1.0%) | 3 (0.6%) | 0.5 |
| T165 | 42 (6.2%) | 47 (8.6%) | 0.1 |
| K166 | 85 (13%) | 68 (12%) | >0.9 |
| I167 | 0 (0%) | 1 (0.2%) | 0.4 |
| E169 | 28 (4.1%) | 26 (4.8%) | 0.6 |
| P170 | 2 (0.3%) | 1 (0.2%) | >0.9 |
| F171 | 5 (0.7%) | 6 (1.1%) | 0.6 |
| R172 | 1 (0.1%) | 1 (0.2%) | >0.9 |
| K173 | 635 (94%) | 515 (94%) | 0.5 |
| Q174 | 353 (52%) | 290 (53%) | 0.7 |
| N175 | 8 (1.2%) | 10 (1.8%) | 0.3 |
| P176 | 3 (0.4%) | 5 (0.9%) | 0.5 |
| D177 | 527 (78%) | 431 (79%) | 0.6 |
| I178 | 171 (25%) | 112 (21%) | 0.05 |
| V179 | 49 (7.2%) | 46 (8.4%) | 0.4 |
| I180 | 1 (0.1%) | 0 (0%) | >0.9 |
| Y183 | 1 (0.1%) | 1 (0.2%) | >0.9 |
| D186 | 0 (0%) | 1 (0.2%) | 0.4 |
| Y188 | 1 (0.1%) | 0 (0%) | >0.9 |
| V189 | 10 (1.5%) | 5 (0.9%) | 0.4 |
| D192 | 0 (0%) | 2 (0.4%) | 0.2 |
| L193 | 0 (0%) | 3 (0.6%) | 0.09 |
| E194 | 9 (1.3%) | 3 (0.6%) | 0.2 |
| I195 | 15 (2.2%) | 12 (2.2%) | >0.9 |
| G196 | 78 (12%) | 76 (14%) | 0.2 |
| Q197 | 29 (4.3%) | 24 (4.4%) | >0.9 |
| R199 | 1 (0.1%) | 1 (0.2%) | >0.9 |
| T200 | 657 (97%) | 517 (95%) | 0.07 |
| K201 | 8 (1.2%) | 3 (0.6%) | 0.4 |
| I202 | 100 (15%) | 77 (14%) | 0.8 |
| E203 | 14 (2.1%) | 18 (3.3%) | 0.2 |
| E204 | 64 (9.4%) | 53 (9.7%) | 0.9 |
| L205 | 0 (0%) | 1 (0.2%) | 0.4 |
| R206 | 1 (0.1%) | 3 (0.6%) | 0.3 |
| Q207 | 665 (98%) | 526 (97%) | 0.09 |
| H208 | 2 (0.3%) | 0 (0%) | 0.5 |
| L210 | 5 (0.7%) | 4 (0.7%) | >0.9 |
| R211 | 514 (76%) | 428 (79%) | 0.3 |
| W212 | 0 (0%) | 2 (0.4%) | 0.2 |
| G213 | 0 (0%) | 1 (0.2%) | 0.4 |
| F214 | 134 (20%) | 104 (19%) | 0.8 |
| D218 | 1 (0.1%) | 1 (0.2%) | >0.9 |
| K220 | 1 (0.1%) | 0 (0%) | >0.9 |
| K223 | 1 (0.1%) | 2 (0.4%) | 0.6 |
| E224 | 3 (0.4%) | 3 (0.6%) | >0.9 |
| L228 | 7 (1.0%) | 5 (0.9%) | 0.8 |
| G231 | 1 (0.1%) | 1 (0.2%) | >0.9 |
| E233 | 1 (0.1%) | 1 (0.2%) | >0.9 |
| P236 | 1 (0.1%) | 0 (0%) | >0.9 |
| D237 | 2 (0.3%) | 1 (0.2%) | >0.9 |
| K238 | 7 (1.0%) | 9 (1.7%) | 0.3 |
| W239 | 5 (0.7%) | 8 (1.5%) | 0.2 |
| T240 | 3 (0.4%) | 1 (0.2%) | 0.6 |
| V241 | 5 (0.7%) | 6 (1.1%) | 0.6 |
| Q242 | 10 (1.5%) | 5 (0.9%) | 0.4 |
| P243 | 41 (6.0%) | 33 (6.1%) | >0.9 |
| I244 | 20 (2.9%) | 11 (2.0%) | 0.3 |
| V245 | 286 (42%) | 229 (42%) | >0.9 |
| L246 | 5 (0.7%) | 2 (0.4%) | 0.5 |
| P247 | 17 (2.5%) | 15 (2.8%) | 0.8 |
| E248 | 16 (2.4%) | 6 (1.1%) | 0.1 |
| K249 | 1 (0.1%) | 4 (0.7%) | 0.2 |
| D250 | 3 (0.4%) | 2 (0.4%) | >0.9 |
| S251 | 0 (0%) | 1 (0.2%) | 0.4 |
| W252 | 1 (0.1%) | 0 (0%) | >0.9 |

***p<0.05; **p<0.01; ***p<0.001** by Fisher’s exact test

**Supplementary Table 3. Comparisons of rates of virologic failure during efavirenz-based-ART by prior PROMISE antepartum component treatment randomization**

| **Prior PROMISE Antepartum**  **Treatment Arm Randomization** | **EFV-ART Virologic Failure**  n/N (%) | **Odds Ratio (95% CI)** |
| --- | --- | --- |
| ZDV Alone (+ sdNVP+TDF/FTC tail) | 76/529 (14.4) | Reference |
| ZDV+3TC+LPV/r | 89/526 (16.9) | 1.21 (0.87, 1.69) |
| TDF+FTC+LPV/r | 24/177 (13.6) | 0.94 (0.57-1.53) |

Note: One woman was excluded from this analysis as she was enrolled after delivery.

Abbreviations: EFV=efavirenz, ZDV=zidovudine, sdNVP=single-dose nevirapine, FTC=emtricitabine, TDF=tenofovir, LPV/r=ritonavir-boosted lopinavir, 3TC=lamivudine, CI=confidence interval

**Supplementary Table 4. Comparisons between rates of virologic failure by pre-EFV HIV drug resistance genotype in women randomized to antepartum TDF+FTC+LPV/r, a post-hoc case-control study**

| Genotyping Method | Pretreatment Genotype | Cases  Virologic Failure  (N = 24) | Controls  ART-Suppression  (N=80) | Unadjusted OR  (95% CI) | p-value |
| --- | --- | --- | --- | --- | --- |
| **Sanger Sequencing**  (≥20% Mutant Cut-Off) | Wild-type | 15 (62.5) | 71 (88.7) | *Reference* |  |
|  | Any Resistance | 8 (33.3) | 9 (11.3) | **4.13 (1.39, 12.3)*** | **0.011** |
|  | NRTI Resistance only | 0 | 0 |  |  |
|  | Single NNRTI Resistance | 8 | 9 | **4.13 (1.39, 12.3)*** | **0.011** |
|  | Multiple NNRTI Resistance | 0 | 0 |  |  |
|  | ≥1 NRTI & ≥1 NNRTI | 0 | 0 |  |  |
|  | Not Sequenced | 1 (4.2) | 0 (-) | N/A |  |
| **Illumina Sequencing**  (≥20%) Mutant Cut-Off) | Wild-type | 14 (58.3) | 69 (86.2) | *Reference* |  |
|  | Any Resistance | 8 (33.3) | 10 (12.5) | **3.88 (1.32, 11.4)*** | **0.014** |
|  | NRTI Resistance only | 0 | 0 |  |  |
|  | Single NNRTI Resistance | 8 | 10 | **3.88 (1.32, 11.4)*** | **0.014** |
|  | Multiple NNRTI Resistance | 0 | 0 |  |  |
|  | ≥1 NRTI & ≥1 NNRTI | 0 | 0 |  |  |
|  | Not Sequenced | 2 (8.3) | 1 (1.3) | N/A |  |
| **Illumina Sequencing**  (≥5%) Mutant Cut-Off) | Wild-type | 13 (54.2) | 68 (85.0) | *Reference* |  |
|  | Any Resistance | 9 (37.5) | 11 (13.8) | **4.19 (1.47, 12.0)**** | **0.008** |
|  | NRTI Resistance only | 0 | 0 |  |  |
|  | Single NNRTI Resistance | 8 | 10 | **4.11 (1.39, 12.2)*** | **0.011** |
|  | Multiple NNRTI Resistance | 1 | 1 | 5.07 (0.39, 66.2) | 0.2 |
|  | ≥1 NRTI & ≥1 NNRTI | 0 | 0 |  |  |
|  | Not Sequenced | 2 (8.3) | 1 (1.2) | N/A |  |
| **Illumina Sequencing**  (≥1%) Mutant Cut-Off) | Wild-type | 13 (54.2) | 63 (78.8) | *Reference* |  |
|  | Any Resistance | 9 (37.5) | 16 (20.0) | 2.71 (0.99, 7.32) | 0.052 |
|  | NRTI Resistance only | 0 | 1 | 1.57 (0.01, 31.1) | 0.8 |
|  | Single NNRTI Resistance | 7 | 9 | **3.71 (1.19, 11.5)*** | **0.025** |
|  | Multiple NNRTI Resistance | 2 | 5 | 2.14 (0.35, 10.0) | 0.4 |
|  | ≥1 NRTI & ≥1 NNRTI | 0 | 1 | 1.57 (0.01, 31.1) | 0.8 |
|  | Not Sequenced | 2 (8.3) | 1 (1.2) | N/A |  |

**Supplementary Table 5. Comparisons of rates of virologic failure by pre-EFV HIV drug resistance genotype (using Stanford GSS ≥10 to EFV, TDF, XTC) in women randomized to antepartum TDF+FTC+LPV/r, a post-hoc case-control study**

| Genotyping Method | Pretreatment Genotype^a^ | Cases  Virologic Failure  (N = 24) | Controls  ART-Suppression  (N=80) | Unadjusted OR  (95% CI) | p-value |
| --- | --- | --- | --- | --- | --- |
| **Sanger Sequencing**  (≥20% Mutant Cut-Off) | Wild-type | 12 (50.0) | 67 (83.8) | *Reference* |  |
|  | Any Resistance | 11 (45.8) | 13 (16.2) | **4.60 (1.71, 12.6)**** | **0.003** |
|  | NRTI Resistance only | 0 | 0 | - | - |
|  | Single NNRTI Resistance | 8 | 13 | **3.40 (1.17, 9.75)*** | **0.025** |
|  | Multiple NNRTI Resistance | 2 | 0 | **27.0 (2.04, 3813)*** | **0.012** |
|  | ≥1 NRTI & ≥1 NNRTI | 1 | 0 | 16.2 (0.82, 2421) | 0.07 |
|  | Not Sequenced | 1 (4.2) | 0 (-) | N/A |  |
| **Illumina Sequencing**  (≥20%) Mutant Cut-Off) | Wild-type | 11 (45.8) | 67 (83.7) | *Reference* |  |
|  | Any Resistance | 11 (45.8) | 12 (15.0) | **5.40 (1.97, 15.3)**** | **0.001** |
|  | NRTI Resistance only | 0 | 0 | - | - |
|  | Single NNRTI Resistance | 9 | 13 | **4.46 (1.55, 12.9)**** | **0.006** |
|  | Multiple NNRTI Resistance | 2 | 0 | **29.3 (2.21, 4152)*** | **0.010** |
|  | ≥1 NRTI & ≥1 NNRTI | 0 | 0 | - | - |
|  | Not Sequenced | 2 (8.4) | 1 (1.3) | N/A |  |
| **Illumina Sequencing**  (≥5%) Mutant Cut-Off) | Wild-type | 10 (41.7) | 66 (82.5) | *Reference* |  |
|  | Any Resistance | 12 (50.0) | 13 (16.3) | **5.86 (2.17, 16.5)***** | **<0.001** |
|  | NRTI Resistance only | 0 | 0 | - | - |
|  | Single NNRTI Resistance | 9 | 12 | **4.81 (1.66, 14.2)**** | **0.004** |
|  | Multiple NNRTI Resistance | 3 | 1 | **14.8 (2.18, 164)**** | **0.006** |
|  | ≥1 NRTI & ≥1 NNRTI | 0 | 0 | - | - |
|  | Not Sequenced | 2 (8.3) | 1 (1.2) | N/A |  |
| **Illumina Sequencing**  (≥1%) Mutant Cut-Off) | Wild-type | 10 (41.7) | 63 (78.7) | *Reference* |  |
|  | Any Resistance | 12 (50.0) | 16 (20.0) | **4.58 (1.73, 12.5)**** | **0.002** |
|  | NRTI Resistance only | 0 | 1 | 2.02 (0.01, 40.5) | 0.7 |
|  | Single NNRTI Resistance | 9 | 9 | **6.05 (2.00, 18.9)**** | **0.002** |
|  | Multiple NNRTI Resistance | 3 | 6 | 3.26 (0.69, 13.6) | 0.13 |
|  | ≥1 NRTI & ≥1 NNRTI | 0 | 0 | - | - |
|  | Not Sequenced | 2 (8.3) | 1 (1.3) | N/A |  |

^a^Interpreted by Stanford HIV Drug Resistance database (version 8.9-1) with “wild-type” defined as GSS <10 and “any resistance mutations” defined as GSS ≥10 for individuals mutations to EFV, TDF, or XTC.

**Supplementary Table 6. Comparison of women with virologic failure vs. ART-suppression during efavirenz-based-ART using alternative definition of HIV pretreatment drug resistance genotype (GSS ≥10 to EFV, TDF, 3TC/FTC)**

|  |  |  |  | **Logistic Regression Model of**  **Virologic Failure on EFV-ART** | |
| --- | --- | --- | --- | --- | --- |
|  | **N** | **Virologic Failure** (n=189) | **ART-Suppressed** (n=1044) | **Unadjusted OR**  **(95% CI)** | **Adjusted OR**  **(95% CI)** |
| **Clinical Data - Median (Interquartile Range)** | **1,233** |  |  |  |  |
| Age in Years (analyzed per 5 years) |  | 24 (21-28) | 26 (23-30) | **0.66 (0.56, 0.78)***** | **0.75 (0.61, 0.90)**** |
| CD4 Count at PROMISE Entry in cells/uL (analyzed per 50 cells/uL) |  | 500 (431-623) | 510 (426-632) | 1.00 (0.96, 1.04) | 1.01 (0.96, 1.06) |
| HIV RNA Load at PROMISE Entry in log10copies/mL |  | 4.03 (3.58-4.56) | 3.90 (3.45-4.40) | **1.33 (1.06, 1.67)*** | 0.90 (0.66, 1.24) |
| HIV RNA Load at EFV Initiation in log10copies/mL |  | 3.95 (3.53-4.62) | 3.85 (3.29-4.44) | **1.32 (1.08, 1.62)**** | 1.12 (0.84, 1.49) |
| Time Between Delivery & EFV Initiation in Weeks (analyzed per 26 weeks) |  | 110 (66.0-145.0) | 113 (63.8-153.0) | 0.98 (0.92, 1.06) | 0.96 (0.86, 1.06) |
| Time Between Last Unsuppressed Visit & EFV Initiation in Weeks (analyzed per 4 weeks) |  | 0.6 (0.0-9.1) | 1.3 (0.0-26.4) | **0.96 (0.94, 0.98)***** | **0.96 (0.93, 0.98)***** |
| Time on EFV in Weeks |  | 55.6 (36.4, 78.9) | 58.0 (43.3, 82.6) | 1.00 (0.99, 1.00) | 1 (0.99, 1.00) |
| **Clinical Site^a^** | **1,233** |  |  |  |  |
| Site A | 220 | 25 (11.4%) | 195 (88.6%) | *Reference* | *Reference* |
| Site B | 206 | 48 (23.3%) | 158 (76.7%) | **2.35 (1.40, 4.0)**** | **2.69 (1.51, 4.92)***** |
| Site C | 147 | 38 (25.9%) | 109 (74.1%) | **2.70 (1.56, 4.73)***** | **2.62 (1.39, 5.01)**** |
| Site D | 152 | 7 (4.6%) | 145 (95.4%) | **0.40 (0.16, 0.88)*** | **0.43 (0.17, 1.00)*** |
| Site E | 121 | 12 (9.9%) | 109 (90.1%) | 0.88 (0.42, 1.76) | 1.25 (0.52, 2.83) |
| Site F | 127 | 16 (12.6%) | 111 (87.4%) | 1.13 (0.58, 2.18) | 1.23 (0.58, 2.53) |
| Sites G-N^b^ | 260 | 43 (16.5%) | 217 (83.5%) | 1.53 (0.91, 2.62) | 1.53 (0.86, 2.80) |
| **HIV RNA Load at Delivery** | **1,200^c^** |  |  |  |  |
| HIV RNA load ≤400 copies/mL | 658 | 72 (10.9%) | 586 (89.1%) | *Reference* | *Reference* |
| HIV RNA load >400 copies/mL | 542 | 113 (20.8%) | 429 (79.2%) | **2.14 (1.56, 2.95)***** | **2.71 (1.76, 4.21)***** |
| **Hepatitis B Virus Test Result** | **1,233** |  |  |  |  |
| HBV negative | 1,191 | 188 (15.8%) | 1,003 (84.2%) | *Reference* | *Reference* |
| HBV positive | 42 | 1 (2.4%) | 41 (97.6%) | **0.19 (0.02, 0.72)*** | **0.17 (0.02, 0.68)**** |
| **PROMISE Antepartum Treatment Regimen** | **1,232^d^** |  |  |  |  |
| Triple ARV (ZDV+3TC+LPV/r) | 526 | 89 (16.9%) | 437 (83.1%) | *Reference* | *Reference* |
| Triple ARV (TDF+FTC+LPV/r) | 177 | 24 (13.6%) | 153 (86.4%) | 0.78 (0.47, 1.25) | 0.69 (0.38, 1.21) |
| ZDV Monotherapy (+ sdNVP+TDF+FTC tail) | 529 | 76 (14.4%) | 453 (85.6%) | 0.82 (0.59, 1.15) | **0.48 (0.31, 0.76)**** |
| **PROMISE Postpartum Treatment Regimen** | **1,013^e^** |  |  |  |  |
| Maternal Triple ARV (TDF+FTC+LPV/r) | 423 | 72 (17.0%) | 351 (83.0%) | *Reference* | *Reference* |
| No Maternal Treatment | 590 | 95 (16.1%) | 495 (83.9%) | 0.93 (0.67, 1.31) | 0.68 (0.46, 1.00) |
| **HIV Genotype at EFV Initiation^f^** | **1,223^g^** |  |  |  |  |
| Wild-type | 1,017 | 154 (17.7%) | 863 (82.3%) | *Reference* | *Reference* |
| Any Resistance Mutations^h^ | 206 | 34 (18.6%) | 172 (81.4%) | 1.12 (0.74, 1.66) | 1.02 (0.64, 1.60) |
| NRTI Mutation(s) Only |  | 0 | 5 | 0.51 (0.00, 4.52) | 0.13 (0.00, 1.43) |
| K103N Only |  | 15 | 76 | 1.13 (0.62, 1.95) | 1.22 (0.61, 2.31) |
| Y181C Only |  | 0 | 7 | 0.37 (0.00, 3.08) | 0.27 (0.00, 2.34) |
| G190A Only |  | 0 | 5 | 0.51 (0.00, 4.52) | 0.38 (0.00, 4.24) |
| Other Single NNRTI Mutation |  | 8 | 61 | 0.77 (0.34, 1.53) | 0.76 (0.33, 1.61) |
| ≥2 NNRTI Mutations |  | 5 | 18 | 1.66 (0.57, 4.11) | 1.24 (0.40, 3.34) |
| ≥1 NRTI & ≥1 NNRTI Mutation |  | 6 | 0 | **72.7 (8.52, 9,493)***** | **57.4 (5.81, 7,743)***** |

***p<0.05; **p<0.01; ***p<0.001** by unadjusted or adjusted logistic regression with Firth’s bias reduction

^a^Overall effect of clinical site on adjusted model of virologic failure was significant (p<0.001), so pairwise comparisons were performed with Clinical site A – which had the most participants enrolled – as the reference group.

^b^Sites with fewer than 100 participants enrolled were aggregated.

^c^Thirty-three participants did not have HIV RNA viral load measurements within 14 days of delivery; four who failed on EFV-ART and 29 who suppressed on EFV-ART.

^d^One participant enrolled after delivery and was not randomized in Antepartum Component of PROMISE.

^e^Comparison only included the women who were randomized after delivery in PROMISE, either to the Postpartum Component or directly to the Maternal Health Component, to evaluate the effect of triple ARV versus no treatment on virologic failure on EFV-ART. Women who were not randomized after delivery (observational follow-up only) were excluded from this analysis (n=220) as this group did not have a uniform treatment assignment.

^f^Two separate models were generated to examine the effects of genotype; one to compare any drug resistance detected vs. wild-type and the other to compare each mutational category (n=7) to wild-type.

^g^Ten samples did not amplify and thus have no genotype.

^h^Pretreatment HIV drug resistance genotype defined by Sanger sequencing of protease and reverse transcriptase regions of *pol* interpreted by Stanford HIV Drug Resistance database (version 8-9.1) with “wild-type” defined as GSS <10 and “any resistance mutations” defined as GSS ≥10 for individuals mutations to EFV, TDF, or 3TC/FTC.

Abbreviations: GSS = genotypic susceptibility score, OR = odds ratio, CI = confidence interval, IQR = interquartile range, EFV = efavirenz, ARV = antiretroviral, ZDV = zidovudine, 3TC = lamivudine, TDF = tenofovir, FTC = emtricitabine, sdNVP = single-dose nevirapine, NRTI = nucleoside reverse transcriptase inhibitor, NNRTI = non-nucleoside reverse transcriptase inhibitor

**Supplementary Table 7. Comparison of women with virologic failure vs. ART-suppression during efavirenz-based-ART within prior PROMISE antepartum treatment arms using alternative definition of pretreatment HIV drug resistance genotype (GSS ≥10 to EFV, TDF, 3TC/FTC)**

|  | **Antepartum ZDV Monotherapy** | | | **Antepartum ZDV+3TC+LPV/r** | | | **Antepartum TDF+FTC+LPV/r** | | |
| --- | --- | --- | --- | --- | --- | --- | --- | --- | --- |
|  | **Virologic**  **Failure** (n=76) | **ART-**  **Suppressed** (n=453) | **Unadjusted OR**  **(95% CI)** | **Virologic**  **Failure^a^** (n=89) | **ART-**  **Suppressed** (n=437) | **Unadjusted OR**  **(95% CI)** | **Virologic**  **Failure^a^** (n=24) | **ART-**  **Suppressed** (n=153) | **Unadjusted OR**  **(95% CI)** |
| **Clinical Data – Median, (IQR)** |  |  |  |  |  |  |  |  |  |
| Age in Years (analyzed per 5 years) | 24 (21, 27) | 26 (23, 30) | **0.65 (0.49, 0.84)**** | 24 (21, 28) | 27 (23, 31) | **0.67 (0.53, 0.85)***** | 22.50 (20.8, 28) | 26 (23, 30) | 0.67 (0.41, 1.05) |
| HIV RNA Load at PROMISE Entry in log10copies/mL | 3.95  (3.56, 4.56) | 3.88  (3.47, 4.35) | 1.30 (0.90, 1.87) | 4.12  (3.70, 4.60) | 3.91  (3.42, 4.45) | **1.44 (1.03, 2.00)*** | 4.24  (3.54, 4.44) | 3.99  (3.57, 4.42) | 1.09 (0.58, 2.00) |
| HIV RNA Load at EFV Initiation in log10copies/mL | 4.02  (3.64, 4.74) | 3.97  (3.30, 4.51) | **1.43 (1.06, 1.94)*** | 3.82  (3.46, 4.57) | 3.74  (3.28, 4.37) | 1.23 (0.90, 1.69) | 3.96  (3.51, 4.54) | 3.91  (3.36, 4.45) | 1.39 (0.77, 2.53) |
| Time Between Delivery & EFV Initiation in Weeks (analyzed per 26 weeks) | 4.22  (2.73, 5.66) | 4.73  (2.85, 6.15) | 0.94 (0.84, 1.05) | 4.77  (2.54, 5.92) | 4.69  (2.96, 6.23) | 0.97 (0.87, 1.08) | 2.88  (2.41, 4.01) | 2.42  (1.46, 3.35) | 1.25 (0.96, 1.63) |
| Time Between Last Unsuppressed Visit & EFV Initiation in Weeks (analyzed per 4 weeks) | 0.04  (0, 0.94) | 0.29  (0, 6.21) | **0.96 (0.92, 0.99)**** | 0.21  (0, 3.11) | 0.29  (0, 7.29) | **0.97 (0.95, 1.00)*** | 0.25  (0, 3.54) | 0.71  (0, 6.18) | 0.96 (0.88, 1.02) |
| **HIV RNA Load at Delivery** |  |  |  |  |  |  |  |  |  |
| HIV RNA load ≤400 copies/mL | 12 (15.8%) | 107 (23.6%) | *Reference* | 49 (55.1%) | 364 (83.3%) | *Reference* | 11 (45.8%) | 115 (75.2%) | *Reference* |
| HIV RNA load >400 copies/mL | 62 (81.6%) | 324 (71.5%) | 1.66 (0.90, 3.29) | 39 (43.8%) | 68 (15.6%) | **4.25 (2.59, 6.95)***** | 12 (50%) | 36 (23.5%) | **3.44 (1.42, 8.44)**** |
| **Hepatitis B Virus Test Result** |  |  |  |  |  |  |  |  |  |
| HBV negative | 76 (100%) | 439 (96.9%) | *Reference* | 89 (100%) | 425 (97.3%) | *Reference* | 23 (95.8%) | 138 (90.2%) | *Reference* |
| HBV positive | 0 (0%) | 14 (3.1%) | 0.2 (0.00, 1.51) | 0 (0%) | 12 (2.8%) | 0.19 (0.00, 1.47) | 1 (4.17%) | 15 (9.80%) | 0.57 (0.06, 2.48) |
| **HIV Genotype at EFV Initiation^a,b,c^** |  |  |  |  |  |  |  |  |  |
| Wild-type | 67 (88.2%) | 366 (81.7%) | *Reference* | 75 (84.3%) | 367 (84.4%) | *Reference* | 12 (52.2%) | 129 (85.4%) | *Reference* |
| Any Resistance Mutations | 9 (11.8%) | 82 (18.3%) | 0.63 (0.29, 1.23) | 14 (15.7%) | 68 (15.6%) | 1.03 (0.54, 1.87) | 11 (47.8%) | 22 (14.6%) | **5.30 (2.10,13.4)***** |
| NRTI Mutation(s) Only | 0 | 3 | 0.78 (0.01, 8.13) | 0 | 2 | 0.97 (0.01, 12.1) |  |  | -- |
| K103N Only | 5 | 37 | 0.8 (0.28, 1.87) | 6 | 30 | 1.04 (0.39, 2.36) | 4 | 9 | **4.91 (1.28, 16.9)*** |
| Y181C Only | 0 | 2 | 1.09 (0.01, 13.5) | 0 | 3 | 0.7 (0.01, 7.28) | 0 | 2 | 2.07 (0.01, 27.4) |
| G190A Only | 0 | 2 | 1.09 (0.01, 13.5) | 0 | 1 | 1.62 (0.01, 30.7) | 0 | 2 | 2.07 (0.01, 27.4) |
| Other Single NNRTI Mutation | 1 | 33 | **0.24 (0.03, 0.94)*** | 3 | 19 | 0.87 (0.23, 2.50) | 4 | 9 | **4.91 (1.28, 16.9)*** |
| ≥2 NNRTI Mutations | 2 | 5 | 2.47 (0.44, 10.5) | 1 | 13 | 0.54 (0.06, 2.26) | 2 | 0 | **51.8 (3.9, 7,303)**** |
| ≥1 NRTI & ≥1 NNRTI Mutation | 1 | 0 | 16.3 (0.86, 2,387) | 4 | 0 | **43.8 (4.6, 5,828)***** | 1 | 0 | **31.1 (1.57, 4,636)*** |

***p<0.05; **p<0.01; ***p<0.001** by unadjusted logistic regression with Firth’s bias reduction

^a^Interpreted by Stanford HIV Drug Resistance database (version 8.9-1) with “wild-type” defined as GSS <10 and “any resistance mutations” defined as GSS ≥10 for individuals mutations to EFV, TDF, or 3TC/FTC.

^b^Two separate models were generated to examine the effects of genotype; one to compare any drug resistance detected vs. wild-type and the other to compare each mutational category (n=7) to wild-type.

^c^Samples without genotype (n=10) did not amplify.

Abbreviations: OR = odds ratio, CI = confidence interval, IQR = interquartile range, EFV = efavirenz, ARV = antiretroviral, ZDV = zidovudine, 3TC = lamivudine, TDF = tenofovir, FTC = emtricitabine, sdNVP = single-dose nevirapine, NRTI = nucleoside reverse transcriptase inhibitor, NNRTI = non-nucleoside reverse transcriptase inhibitor, -- = not analyzed
